# Sentinel Lymph Node Mapping with Indirect Lymphangiography for Canine Mast Cell Tumour

**DOI:** 10.3390/vetsci9090484

**Published:** 2022-09-08

**Authors:** Andrea De Bonis, Francesco Collivignarelli, Andrea Paolini, Ilaria Falerno, Valentina Rinaldi, Roberto Tamburro, Amanda Bianchi, Rossella Terragni, Jacopo Gianfelici, Paolo Frescura, Giulia Dolce, Eleonora Pagni, Roberta Bucci, Massimo Vignoli

**Affiliations:** 1 Faculty of Veterinary Medicine, University of Teramo, 64100 Teramo, Italy; 2 Clinica Veterinaria Pet Care, 40133 Bologna, Italy; 3 Clinica Veterinaria Val Musone, 60027 Osimo, AN, Italy; 4 Clinica Veterinaria Pescara Nord, 65124 Pescara, Italy; 5 Policlinico Veterinario Roma Sud, 00173 Roma, Italy; 6 Clinica Veterinaria Etruria 24H, 53035 Siena, Italy

**Keywords:** mast cell tumour, indirect lymphangiography, sentinel lymph node, dog

## Abstract

**Simple Summary:**

Mast cell tumour (MCT) is a common cutaneous and subcutaneous neoplasia in dogs. Recent studies describe that sentinel lymph node (SLN) assessment is more specific to stage MCT, while regional lymph node (RLN) evaluation is not as specific. SNL is the first site of drainage of a tumour and the first metastatic site in several tumours. The study aims to evaluate the SLN drainage mapping of MCT with indirect lymphography in dogs. The second objective of the study is to compare the SLN to the RLN. Survey radiographs followed by an indirect lymphography were obtained for SLN mapping. Twenty-six dogs with 29 MCTs were included. SLNs were detectable in 26 MCTs and radiographic indirect lymphangiography with Lipiodol was able to detect at least one SLN in 90% of MCTs in dogs. In conclusion, radiographic indirect lymphangiography with Lipiodol is a feasible technique to map SLNs and its draining system in MCTs. The lymph drainage pattern of the MCTs may be different for each MCT and more than one SLN can be involved.

**Abstract:**

Mast cell tumour (MCT) is a common cutaneous and subcutaneous neoplasia in dogs. It can metastasise to lymph nodes (LNs), and this adversely affects the prognosis and treatment. The study aims to evaluate the SLN mapping of MCTs with radiographic indirect lymphography. Dogs that underwent clinical staging were prospectively enrolled. Lipiodol was injected around the MCT or the surgical scar. After 24 h, LNs that picked up contrast were radiographically assessed. Twenty-six dogs with 29 MCTs were included. MCTs were confirmed histologically, while SLNs were evaluated either by cytology and/or histology. SLNs were detectable in 23 dogs with 26 MCTs. Lymphatic vessels were visible in 19 MCTs. In nine MCTs, at least two SLNs picked up contrast. In particular, seven MCTs involved two SLNs, and two MCTs involved three different SLNs. In nine MCTs, at least a SLN was metastatic. This study indicates that the lymph drainage pattern of the MCTs may be different for each MCT, and more than one SLN can be involved. Indirect lymphangiography with Lipiodol allowed the detection of the SLN in 90% of MCTs. This provided clinically relevant information to remove the LN and stage the patient.

## 1. Introduction

Mast cell tumour (MCT) is a common malignant cutaneous and subcutaneous neoplasia in dogs [1,2,3,4]. It is reported to be 16–21% of all canine integumentary tumours [1,2,3,4]. MCT can metastasise to lymph nodes (LNs), and this adversely affects the prognosis and treatment [1]. The World Health Organisation (WHO) reported a classification to differentiate the staging of MCTs, to better evaluate the prognosis and treatment of MCTs in veterinary medicine [5]. MCTs with metastatic LNs were ranked as stage II, leading to a worse prognosis and requiring different treatment compared to stage 0 or stage I [5]. Initially, regional lymph node (RLN) assessment was considered the standard care for stage MCT [6]. More recent studies describe that sentinel lymph node (SLN) assessment is more accurate to stage MCT, while RLN evaluation is not as accurate [6,7,8,9]. SNL is the first site of drainage of a tumour and in several tumours the first metastatic site [7]. Several studies report a different percentage between SLN and RLN, in particular in Laspley et al. in 27% of dogs, in Worley et al. in 42% of dogs, and in Ferrari et al. in 63% of dogs. [6,7,8,9].

In both the human and veterinary literature, different techniques are reported to help detection of SLN [9,10,11,12,13,14]. SLN mapping for MCT in veterinary medicine has been evaluated with several techniques such as contrast-enhanced ultrasound [10], lymphoscintigraphy [7,8], and computed tomography (CT) indirect lymphangiography [15]. In human medicine, lymphoscintigraphy [16] is the gold standard for mapping SLN, however, in veterinary medicine, only a few centres have the instrumentations to perform it [10]. CT indirect lymphangiography has recently been widely used for different tumour staging [10,13]. There are several disadvantages related to the techniques mentioned above, such as the requirement of general anaesthesia, high cost, and, finally, multiple scans must be performed to correctly detect the lymphatic vessels and SLN [15].

There are only few studies about indirect lymphangiography performed with radiographs. A recent publication mentions the utility of this technique for SLN draining in mammary tumours [12]. Advantages are the wide availability of radiology, the safety of the contrast media injected at the periphery of the tumour, and the possibility to perform the procedure with the dog awake or mildly sedated [12].

The study aimed to evaluate the SLN drainage mapping of MCTs with indirect lymphography in dogs. The second object of the study was to correlate the SLN to the RLN.

We hypothesised that indirect lymphangiography with the radiographic study would be a feasible technique to detect the SLN of canine MCTs. Our second hypothesis was that SLN would not always coincide with the RLN of the MCTs.

## 2. Materials and Methods

This study protocol N. 10/2020 was approved by Committee on Animal Research and Ethics of the Universities of Chieti-Pescara, Teramo, L’Aquila and of the Experimental Zoo prophylactic Institute of Abruzzo-Molise (CEISA), according to the Italian law decree N. 26/2014 and to EU directive 2010/63. The owner signed an informed consent form before the procedure.

This prospective multicentric observational study was conducted from January 2021 to March 2022 at the Veterinary Teaching Hospital of the University of Teramo, veterinary clinic Pet Care, veterinary clinic Val Musone, veterinary clinic Pescara Nord, veterinary clinic Roma Sud, and veterinary clinic Etruria H24.

Dogs were eligible for the study if they were undergoing staging with complete blood work consistent with CBC and chemistry of a histologically diagnosed MCT. The histology was sent to external laboratories, where board-certified pathologists or experienced operators classified the MCT according to both Kiupel’s 2011 [17] and Patnaik’s 1984 [18] grading system [2], while subcutaneous tumours were measured according to the Thompson grading system [19]. Dogs with scars of already excised MCTs were also eligible if there was an incomplete resection of the margins. Dogs were excluded from the study if the RLN was previously removed.

Survey radiographs followed by an indirect lymphography were obtained for SLN mapping. If needed, premedication was always performed intramuscularly (IM) with methadone (semfortan^®^; Eurovet Animal Health, Bladel, The Netherlands) ranging from 0.2–0.3 mg kg^−1^ and dexmedetomidine (Dextroquillan^®^; Fatro, Bologna, Italy) 0.003–0.006 mg kg^−1^, according to anaesthetist clinical evaluation. At the end of the procedures, atipamezole (Antisedan^®^; Vetoquinol, Magny-Vernois, France) was administrated IM at ½ of dexmedetomidine dose.

**Figure 1:**
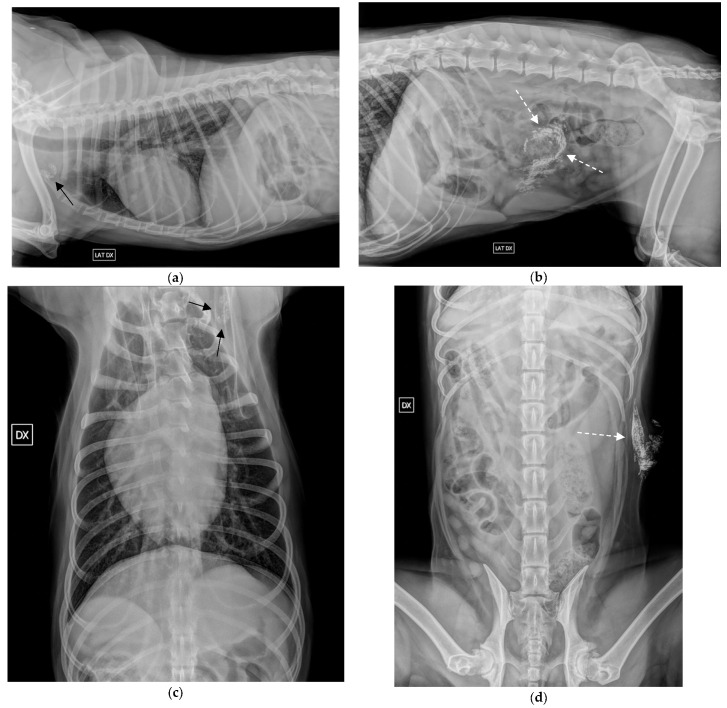
Radiographic indirect lymphangiography 24 h post-Lipiodol of a MCT in the left abdominal wall. (**a**,**c**) right lateral and VD view of the thorax. The left axillary SLN is highlighted by the black arrow; (**b**,**d**) Right lateral and VD view of the abdomen. The Lipiodol was injected at the periphery of the tumour in the left abdominal wall (white dashed arrow).

The radiographic study pre- and post-contrast administration and the injection of contrast medium were performed with the dog awake or mildly sedated according to dog collaboration. If needed, sedation was always performed intramuscularly (IM) with methadone (semfortan^®^; Eurovet Animal Health, Bladel, The Netherlands) ranging from 0.2–0.3 mg kg^−1^ and dexmedetomidine (Dextroquillan^®^; Fatro, Bologna, Italy) 0.003–0.006 mg kg^−1^, according to anaesthetist clinical evaluation. At the end of the procedures, atipamezole (Antisedan^®^; Vetoquinol, Magny-Vernois, France) was administrated IM at ½ of the dexmedetomidine dose. Every radiographic study was performed with at least two orthogonal views, according to the location of the primary tumour and the RLNs. If no SLNs were detected in the expected site, an additional region with the radiographic study was included. Immediately after the radiographic examination, Lipiodol Ultra-Fluid TM (iodized ethyl esters of the fatty acids of poppy seed oil, Guerbet, Aulnay-sous-Bois, France; 480 mg iodine mL^−1^) was injected around the MCT (Figure 1) or the surgical scar. The procedure was performed with a 25 G needle into the subcutis of four quadrants surrounding the tumour, as previously described [12,20]. A slow rate infiltration, 0.2–0.4 mL for each quadrant for a total of 0.8–1.6 mL of contrast medium over 1–2 min, was injected depending on the tumour size [12]. Care was taken to avoid any contrast leakage, or contrast entering the blood vessels or injecting directly into the primary tumour. There are only a few collateral effects reported in human medicine, such as embolism with a 0.12% frequency following lymphangiography, or hypersensitivity reaction that can happen within 60 min of the injection [21,22].

Dogs who were not sedated were monitored to see any possible collateral effects and discharged one hour after lipiodol injection. Otherwise, if the dog underwent sedation, they were carefully monitored and discharged one hour after the end of the sedation and a standing position was obtained. After 24 h, dogs were admitted again and the same radiographic study was repeated; if no SLNs were detected in the expected site additional regions were included in the radiographic study, and the site and number of the SLNs that picked up contrast were radiographically assessed. If visible, the presence of lymphatic vessels was also reported. Each radiographic study was evaluated by a second-year resident ECVDI (A.D.B.) and a board-certified radiologist (M.V.). The two observers independently reviewed the location of SLNs, visibility of lymphatic vessels, and interobserver agreement was assessed. In order to help identification of the location of the LNs, anatomical landmarks were mentioned to better locate the position of the SLNs. In case of disagreement between operators’ assessments, radiologists evaluated images together to find an agreement. The evaluation of if SLN correlated to RLN was divided into 3 groups. Group 1 was “total agreement”, when RLN was the same as the SLN. Group 2 was “partial agreement”, when one SLN coincided with the RLN, but a second SLN did not correspond with the RLN. Group 3 was “no agreement”, when the RLN did not correspond to any SLN. SLNs that were identified through indirect lymphangiography were investigated by either cytology following the Krick et al. classification [23], or were surgically removed and assessed by histological exam according to the Weishaar et al. system, where HN2 and HN3 are considered as metastatic [24] by a board-certified pathologist or an experienced operator.

## 3. Results

### 3.1. MCTs

A total of 27 dogs and 31 MCTs were initially included in the study. A single dog who underwent lipiodol staging at the same time with two different MCTs showed three different SLNs. It was not possible to differentiate if the SLNs were draining one, the other, or both the MCTs, so it was removed from the total count, leaving a total of 26 dogs and 29 MCTs. The population sample consisted, for the majority, of mixed breeds and Labrador, two Maltese and English Setter, while the remaining breeds were each represented by a single dog. The population sample is summarised in Table 1.

The masses were located at the level of head and neck (nine), thorax (six), hindlimbs (six), abdomen (three), forelimbs (two), scrotum (two), and prepuce (one). The location of the MTCs is summarised in Table 2.

Twenty-three dogs included in the study have a single MCT. A single dog included in the study has a surgical scar of a single MCT surgically removed with positive margins. One dog presented with two different MCTs, and one subject presented with three different MCTs during the inclusion.

#### MCTs Histology

The MCTs included in the study were: 8 subcutaneous (scMCT), and 23 cutaneous. Of the cutaneous MCT (cMCT), there were 3 Patnaik grade I–low-grade Kiupel (P1-LK), 16 Patnaik grade II–low-grade Kiupel (P2-LK), and 4 Patnaik grade II–high-grade Kiupel (P2-HK). No grade III Patnaik are presented in the study. The histopathological classification is summarised in Table 3.

### 3.2. SLNs Mapping

**Figure 2:**
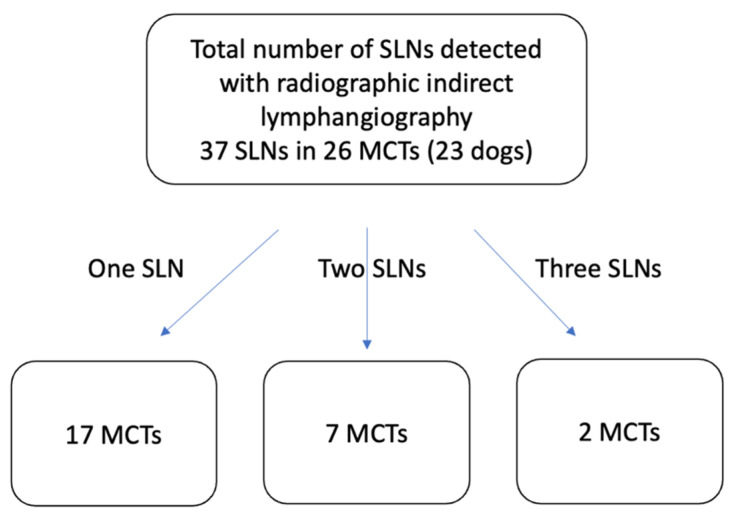
Number of SLNs detected with radiographic indirect lymphangiography. Abbreviations: SLN, sentinel lymph node; MCT, mast cell tumour.

**Figure 3:**
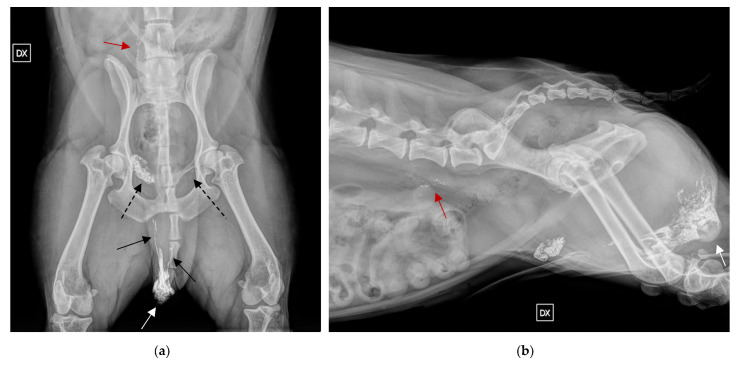
Radiographic indirect lymphangiography 24 h post Lipiodol in two orthogonal views of a MCT in the scrotum with three SLNs: (**a**) VD view of the pelvic region. The Lipiodol was injected at the periphery of the tumour in the scrotum. Lymphatic vessels are visible on both views extending cranially (black arrows), inguinal lymph nodes are highlighted by the (black dashed arrow); (**b**) fight lateral view of the pelvic region. The right medial iliac SLN can be assessed ventrally to L6, pointed by the black arrow, and to the right of L6 in the VD highlighted by the red arrow. The location of the primary MCT in both VD and right lateral is highlighted by the white arrow.

A total of 26 MCTs (90%) and 37 SLNs are correctly identified with indirect lymphangiography (Figure 2). Two cMCTs of different histotypes (2PLK, 2PHK) do not show any SLN with indirect lymphangiography. One scMCT shows just the lymphatic vessels without any SLN. Out of the 26 MCTs that indirect lymphangiography correctly identified the SLN, 17 MCTs show a single SLN, and 9 MCTs present more than one SLN. Among the latter category, seven MCTs present two SLNs and two MCTs present three SLNs with indirect lymphangiography (data summarised in Table 3) (Figure 3). Immediate or delayed side effects are not reported secondary to the injection of Lipiodol in any dog of our study. There is a complete agreement between the observers for the evaluation of SLNs, and for visible lymphatic vessels. We classify 10 SLNs (27%) as in agreement with the RLNs, 7 SLNs (19%) in non-agreement with the RLNs, and 20 SLNs (54%) as in partial agreement with the RLNs. Following the indirect lymphangiography, it is possible to evaluate the lymphatic vessel (Figure 3) (Table 3) in 17 MCTs. Among them, the lymphatic vessel associated with a single SLN in 11 MCTs, with two SLNs in 5 MCTs, three SLNs in 2 MCTs, and only one MCT shows a lymphatic vessel with no evidence of SLN. Otherwise, in 10 MCTs, lymphatic vessels are not visible following indirect lymphangiography. Out of these, five MCTs are associated with one SLN, three MCTs with two SLNs, and two MCTs do not present any SLN.

#### SLNs Cytology/Histology

In Table 3, the MCTs grading is associated with the cytology/histology of the SLNs. A total of 37 SLNs were analysed through one of the two methods mentioned above. Among the 37 SLNs, 12 (32%) are metastatic (10 histologically diagnosed and 2 through cytology). A total of 20/37 SLNs were evaluated through histology after surgery, while the remaining 17/37 SLNs were assessed with cytology [19]. Out of the 20 SLNs who underwent histological, grading is classified as metastatic in 10 SLNs (three HN2 and seven HN3), while 10 SLNs are not metastatic (seven HN0 and three HN1). Based on the cytological exam, only 2 SLNs out of 17 are metastatic, while in the remaining 15 SLNs, no evidence of metastatic disease is identified according to Krick et al. [23].

## 4. Discussion

Radiographic indirect lymphangiography with Lipiodol is able to detect at least a SLN in 90% of primary tumours (26/29 MCTs). The results obtained in our study are in accord with our hypothesis, wherein the technique is feasible and allows the evaluation of SLNs in dogs with MCTs. The second aim was to evaluate the agreement between the RLN and SLN. In our study, 26% (7/26) of MCTs have one SLN that does not agree with the RLN, and 34% (9/26) of MCTs have a partial agreement between SLN and the RLN. These findings agree with recent studies where a different SLN from the RLN was detected, ranging from 20 to 60% of cases, through different SLN mapping techniques in dogs with MCTs [6,7,8,9]. Among the MCTs, 9/26 (34%) present at least two different SLNs and, out of those, seven MCTs present two different SLNs, and two MCTs exhibit three different SLNs. This result shows how multiple SLNs, from different centres, can drain the same MCT. Multiple SLNs are reported for MCTs with other mapping methods. Multiple SLNs always represent at least a different SLN from the RLN. Furthermore, in our study, it is important to note that 34% of MCTs have SLNs that only partially agree with RLN, added to the 26% of MCTs with a SLN that does not agree with a RLN, which leads to a total number of 60% of MCTs that, without this mapping technique, would have been improperly evaluated for the correct SLNs and staging [7,15]. There is a complete interobserver agreement for SLNs location and lymphatic vessels visibility. The results represent the relatively easy assessment of the SLNs thanks to the contrast medium within it, leading even a less experienced observer to properly locate the SLNs, giving rise to greater repeatability of the technique.

SLNs mapping gives fundamental information to better evaluate the prognosis of the patient, because, as reported in the literature, SLN usually is the first site of metastasis in MCTs [7]. Studies mention how the presence of metastatic LNs is considered an important prognostic factor, influencing the treatment and the survival time in MCTs [25]. Furthermore, Horta et al. report a higher risk of death (62%) in patients with tumour recurrence and metastatic LNs in MCTs [3].

The results of this study highlight, even more, the necessity of this method for staging dogs instead of evaluating just the RLN. Without indirect lymphangiography, it would not be possible to correctly detect SLN in some cases.

Lipiodol Ultra-Fluid TM in indirect lymphography was used to map the lymphatic drainage of different tumours such as mammary carcinoma [12,26]. To the best of our knowledge, radiographic indirect lymphangiography with Lipiodol for MCTs was never evaluated.

Lymphatic vessels are visible in 65% (19/29) of MCTs. Lymphatic vessels could be useful to help detect the SLNs if the contrast uptake was weak or not homogenous in SLNs. Lymphatic vessels are currently reported through other methods such as CT indirect lymphography and lymphoscintigraphy [7,15,20], although their presence is not correlated with the histologic grade of the MCTs. In our study, no association is found between the classification of the tumour and the presence of the lymphatic vessels or the histologic/cytologic grade of the SLNs. An interesting finding is that 75% (6/8) of scMCTs are associated with visible lymphatic vessels after contrast administration. An explanation for this finding cannot be found, although four of the six subcutaneous MCTs have metastatic SLN, so it may be possible that more aggressive behaviour of the primary tumour leads to the detection of lymphatic vessels with radiographic indirect lymphangiography. On the other hand, out of the other two subcutaneous MCTs that do not show any lymphatic vessel, one of those also has a metastatic SLN. More studies are needed to evaluate this finding.

In three MCTs, the indirect lymphangiography with Lipiodol does not highlight any SLNs; out of these, a single MCT shows lymphatic vessels without any SLNs. A possible explanation would be the failure of the method, such as the contrast being injected too rapidly leading to rupture of the lymphatic vessels. Moreover, the multicentric nature of the study could lead an untrained operator to perform the procedure improperly, such as a fast rate injection of the contrast medium leading to a rupture of the lymphatic vessels, increasing the chance of failure of the procedure. Another potential cause to consider is macro metastasis in the SLNs and lymphatic system. These conditions, as reported in the literature, compromise the visibility of the lymphatic vessels and SLNs [27]. As reported in the literature, the surgical asportation of the MCT could lead, theoretically, to disruption of the normal lymphatic vasculature, and the consequent failure of the indirect lymphangiography on the surgical scar [7]. In our study, the three MCTs in which indirect lymphangiography is not successful are performed at the periphery of the MCTs. On the contrary, in the only dog included with a surgical scar, the method highlights a SLN that is also metastatic, confirmed with histology.

The advantages of radiographic indirect lymphangiography are the wide availability of the technique, and the possibility to perform the study with the dog awake or mildly sedated [12]. Meanwhile, computed tomography SLN mapping is associated with increased costs, more limited availability, and the need to perform the study under general anaesthesia [28,29]. Computed tomography SLN mapping in MCTs correctly identifies SLN in 60% of different tumour types in a study [27], while our indirect lymphangiography with radiographs has a higher success rate (90%). Another difference between the two techniques is the need to repeat the study for every patient in CT at 1, 3, 6, 9, and 12 min, in order to find the time where the contrast enhancement is more homogenous and a proper assessment of the SLN is possible; on the other hand, we only had to repeat the radiographic study once 24 h after the injection to properly evaluate the SLN. The study mentioned in veterinary medicine with lymphoscintigraphy is able to detect SLNs in 91% of MCTs [7], similar to our result of 90% of SLNs detected with indirect lymphangiography in MCTs. The interesting finding that differs from the paper mentioned above is the type of cases in which the lymphoscintigraphy is not successful. The three MCTs in which the method fails were all performed on a surgical scar in the study by Ferrari et al. [7], while, in our study, the indirect lymphangiography performed around the only surgical scar case included successfully identifies the SLN. In fact, the three MCTs wherein the method fails to detect the SLN reported in our study are performed at the periphery of the primary tumour. Unfortunately, in veterinary medicine, not many facilities have the instrumentation for lymphoscintigraphy. Furthermore, care must be considered while manipulating this radiopharmaceutical to guarantee the safety of the operator [30]. Moreover, radiographic indirect lymphangiography is safe to use, and immediate or delayed side effects are not reported secondary to the injection in any dog in our study.

This study has some limitations. The small size of the sample included in the study and a small number of high-grade Kiupel MCTs. This reflects the incidence of the tumour in the literature, because of the lower prevalence of high-grade Kiupel MCTs in dogs compared to the low-grade Kiupel [25,31,32]. Another limitation is the SLNs evaluation with cytology and histology. The gold standard to properly evaluate the malignancy of the LN is histopathology [24]. No intraoperative procedure, such as blue methylene injection, was performed to help detect the SLN identified through the radiographic study, so it cannot be confirmed that the SLNs sampled with FNAs or surgically removed coincided to the one highlighted through the indirect radiograph. This study aimed to evaluate the mapping SLNs with radiographic indirect lymphangiography in MCTs, and not to compare metastasised SLNs, which is why SLNs evaluated through cytology were included, even though including SLNs through cytology could lead to a false negative reading of non-metastatic SLNs. It is possible that if histopathology was performed in every single SLN, the metastatic rate reported in this study could have been even higher. In the study, 12/37 (32%) SLNs are found to be metastatic. Out of those ten, through histology it is confirmed that seven are HN3 and three are HN2. Considering the MCTs in dogs, there are 9/26 (34%) MCTs with SLNs metastasised either by cytology or histology. This relatively high metastatic rate is reported in the literature [25,33,34,35]. In the cMCT group, there are a total of four MCTs with metastatic SLNs. Out of these, two are P2-HK, one P2-LK, and one P1-LK. If we consider the total number of P2-HK included in the study with a successful indirect lymphangiography, we have 2/3 (66%) of these MCTs with metastatic SLNs, while only 2/16 (12%) MCTs include the P1-LK and P2-LK group with metastatic SLNs. These findings agree with what is reported in the literature, where P2-HK has a more aggressive behaviour compared to P1-LK and P2-LK [1]. In the scMCTs group, 5/7 scMCTs are identified with metastatic SLNs. This is controversial to what is reported in the literature, where a recent study mentions a low grade of metastasis of subcutaneous tumours [25]. Is it possible that scMCTs could have more aggressive behaviour than what is reported in the literature, but further studies are needed to confirm this result.

## 5. Conclusions

In conclusion, radiographic indirect lymphangiography with Lipiodol is a feasible technique to map SLN in MCT. The lymph drainage pattern of the MCTs may be different for each MCT, and more than one SLN can be involved. Few SLNs have a different location from the RLNs of the MCTs. SLNs mapping techniques should become the standard protocol for staging the dog, instead of evaluating RLNs. Detection of SLNs would provide clinically relevant information to remove the LN and stage the dog.

## Figures and Tables

**Table 1:** Population data. Breed and sex.

Breed	Mix	Labrador Retriever	English Setter	Maltese	Other breeds: (*n* = 11) 42%
	(*n* = 7) 27%	(*n* = 4) 15%	(*n* = 2) 8%	(*n* = 2) 8%	
Sex	Male	Female	Neutered Male	Spayed Female	
	(*n* = 9) 34.5%	(*n* = 9) 34.5%	(*n* = 3) 11.5%	(*n* = 5) 19.5%	

Abbreviation: mix, mixed breed; n, number.

**Table 2:** Location of MTCs, RLNs, and SLNs assessed with indirect lymphangiography.

MCT	Location	RLN	SLN Mapping
Case 1	Left stifle	**Left popliteal**	**Left inguinal**
Case 2	Upper left lip	Left mandibular	Left mandibular
Case 3	Left neck	**Left cervical superficial**	**Left medial retropharyngeal**
Case 4	Left prescapular	Left cervical superficial	Left cervical superficial
Case 5	Right caudal thigh	Right inguinal	Right inguinal
Case 6	Right lateral thigh	Right inguinal	Right inguinal
Case 7 *	Left sternum	Left axillary	Left axillary
Case 8	Right sternum	*Right axillary*	*Right axillary* *Left axillary*
Case 9	Right prepuce	Right inguinal	Right inguinal
Case 10	Right abdominal wall	**Right inguinal**	**Right medial iliac**
Case 11	Right stifle	*Right popliteal*	*Right popliteal* *Right medial iliac*
Case 12	Left thorax 11th rib	Right Axillary	/
Case 13	Right ventral neck	Right cervical superficial	Right cervical superficial
Case 14	Right thorax 8th rib	*Right axillary*	*Right axillary* *Right accessory axillary*
Case 15	Left scrotum	Left inguinal	Left inguinal
Case 16	Right pre-scrotum	*Right inguinal*	*Right inguinal* *Left inguinal* *Right medial iliac*
Case 17	Left abdominal wall	**Left inguinal**	**Left axillary**
Case 18	Left ear base	*Left cervical superficial*	*Left cervical superficial* *Left deep cervical*
Case 19	Left thigh	*Left inguinal*	*Left inguinal* *Left medial iliac*
Case 20	Right abdominal wall	**Right inguinal**	**Right accessory axillary**
Case 21	Left ear pinna	*Left cervical superficial*	*Left cervical superficial* *Left mandibular*
Case 22	Left thigh	*Left inguinal*	*Left inguinal* Left popliteal *Left medial iliac*
Case 23	Left shoulder	Left axillary	Left axillary
Case 24	Right ear pinna	*Right cervical superficial*	*Right cervical superficial* *Right deep cervical*
Case 25	Right dorsal neck	**Right cervical superficial**	**Right mandibular**
Case 26	Left gluteus	Left inguinal	/
Case 27	Right neck	**Right cervical superficial**	**Right mandibular**
Case 28	Right neck	Right cervical superficial	/
Case 29	Left axilla	Right axillary	Right axillary

* MCTs of the dogs who underwent indirect lymphangiography at the surgical scar. Abbreviations: MCT, mast cell tumour; RLN, regional lymph node; SLN, sentinel lymph node. In bold are the SLNs that do not agree with RLN, while in italic are the SLNs who partially agree with RLN.

**Table 3:** Histological diagnosis of MCTs, cytology/histology of SLNs compared with the location of SLNs, presence or absence of lymphatic vessels.

MCT	SLNs	Cytology/Histology SLN	Lymphatic Vessel	Histology MCTs
Case 1	Left inguinal	HN2 (Histo)	Y	Subcutaneous
Case 2	Left mandibular	HN0 (Histo)	N	P2-LK
Case 3	Left medial retropharyngeal	Positive (Cyto)	Y	P2-HK
Case 4	Left cervical superficial	HN0 (Histo)	Y	P2-HK
Case 5	Right inguinal	Negative (Cyto)	Y	P2-LK
Case 6	Right inguinal	Negative (Cyto)	Y	P2-LK
Case 7 *	Left axillary	HN3 (Histo)	Y	Subcutaneous
Case 8	Left axillary Right axillary	HN2 (Histo)	N	Subcutaneous
Case 9	Right inguinal	HN0 (Histo)	Y	P2-LK
Case 10	Right medial iliac	Negative (Cyto)	Y	P2-LK
Case 11	Right popliteal Right medial iliac	Negative (Cyto)	Y	P2-LK
Case 12	/	/	N	P1-LK
Case 13	Right cervical superficial	Negative (Cyto)	Y	P2-LK
Case 14	Right axillary Right accessory axillary	Negative (Cyto)	Y	P2-LK
Case 15	Left inguinal	Negative (Cyto)	N	P2-LK
Case 16	Right inguinal Left inguinal Right medial iliac	Negative (Cyto)	Y	P2-LK
Case 17	Left axillary	HN1 (Histo)	N	Subcutaneous
Case 18	Left cervical superficial Left deep cervical	Negative (Cyto)	N	P2-LK
Case 19	Left inguinal Left medial iliac	HN3 (Histo)	Y	P2-LK
Case 20	Right accessory axillary	Negative (Cyto)	N	P2-LK
Case 21	Left cervical superficial Left mandibular	HN1 (Histo)	Y	P2-LK
Case 22	Left inguinal Left popliteal Left medial iliac	HN0 (Histo)	Y	Subcutaneous
Case 23	Left axillary	HN0 (Histo)	Y	P1-LK
Case 24	Right cervical superficial Right deep cervical	HN3 (Histo)	N	P2-HK
Case 25	Right mandibular	Positive (Cyto)	N	P1-LK
Case 26	/	/	N	P2-HK
Case 27	Right mandibular	HN3 (Histo)	Y	Subcutaneous
Case 28	/	/	Y	Subcutaneous
Case 29	Right axillary	HN3 (Histo)	Y	Subcutaneous

* Single dog with indirect lymphangiography performed on the surgical scar. / MCTs with no SLNs highlighted. Abbreviations: MCT, mast cell tumour; SLN, sentinel lymph node; HN, histological grade; P, Patnaik; Histo, histopathology; Cyto, cytology; Y, yes; N, no; P1-LK, Patnaik I–low-grade Kiupel; P2-LK, Patnaik II–low-grade Kiupel; P2-HK, Patnaik II–high-grade Kiupel.

## Data Availability

Not applicable.
